# Supplementary material for: The Prevalence of Cancer Predisposition Syndromes (CPSs) in Children with a Neoplasm: A Cohort Study in a Central and Eastern European Population
Source: Genes (Basel). 2024 Aug 29;15(9):1141. doi: 10.3390/genes15091141 (PMC11431396; doi:10.3390/genes15091141)
Supplement: Supplementary file 1 [file genes-15-01141-s001.zip › genes-3154526-supplementary.pdf]

Table S1: Cancer predisposition syndromes (CPSs) detected in 193 patients according to age at time of neoplasm diagnosis. BWS: Beckwith-Wiedemann Syndrome, CMMRD: Constitutional mismatch repair deficiency Syndrome, FA: Fanconi anemia, feat.: features, HSCR: Hirschsprung disease, HLH: Hemophagocytic lymphohistiocytosis, IHH: Isolated hemihypertrophy, LFS: Li-Fraumeni Syndrome, MEN: Multiple endocrine neoplasia Syndrome, mut.: gene mutation, NBS: Nijmegen breakage Syndrome, NF1: Neurofibromatosis type I, RTPS: Rhabdoid tumor predisposition Syndrome, S.: Syndrome, VHL: Hippel-Lindau Syndrome

**Age at time of cancer diagnosis [m: month(s) or y: year(s)]**	**No. of cases**	**Cancer predisposition syndrome (CPS)**																				
		**BWS**	**BRCA1 mut.**	**CMMRD**	**DICER1 mut.**	**Down S.**	**Edwards' S.**	**FA + BRCA2 mut.**	**Feat. of dysmorphia**	**HSCR**	**HLH**	**IHH**	**IMAGE S.**	**LFS**	**LFS + NBS**	**MEN S.**	**NBS**	**NF1**	**Noonan S.**	**RTPS**	**VHL**	**WAGR S.**
0 to < 1 y	49	5			2	3	1		1			6		5				23		2	1	
0 to < 1 m	11	1				2												7		1		
1 to < 2 m	6					1								1				4				
2 to < 3 m	3						1							1						1		
3 to < 4 m	3																	3				
4 to < 5 m	1								1													
5 to < 6 m	5	2										1		1				1				
6 to < 7 m	4	1										2		1								
7 to < 8 m	3				2													1				
8 to < 9 m	3																	2			1	
9 to < 10 m	5	1										3						1				
10 to < 11 m	2													1				1				
11 to < 12 m	3																	3				
1 to < 2 y	12					3		1			2			4				2				
2 to < 3 y	25	3	1			4				1		1		4				10				1
3 to < 4 y	25	2		2		3					2	4		6			1	5				
4 to < 5 y	14					3						4	1	2				4				
5 to < 6 y	8											2		1				5				
6 to < 7 y	4													1				2	1			
7 to < 8 y	5											1		1		1		2				
8 to < 9 y	2																	2				
9 to < 10 y	7					1								1		1		4				
10 to < 11 y	9								1					2				6				
11 to < 12 y	5					1								2				2				
12 to < 13 y	7					1									1	1		4				
13 to < 14 y	3																	3				
14 to < 15 y	2										1							1				
15 to < 16 y	3															1		2				
16 to < 17 y	7													3			1	3				
17 to < 18 y	6								1					2				3				
**No. in total**	**193**	**10**	**1**	**2**	**2**	**19**	**1**	**1**	**3**	**1**	**5**	**18**	**1**	**34**	**1**	**4**	**2**	**83**	**1**	**2**	**1**	**1**

Table S2: Cancer predisposition syndromes (CPSs) detected in 193 patients according to diagnosed neoplasm defined by ICD-10 classification. In two cases two genetic disorders were detected (FA with BRCA2 gene mutation, LFS with NBS), in three cases multiplied cancers were diagnosed (C49 with C64 in one case, C49 with C92 in two cases). BWS: Beckwith-Wiedemann Syndrome, CMMRD: Constitutional mismatch repair deficiency Syndrome, FA: Fanconi anemia, HSCR: Hirschsprung disease, HLH: Hemophagocytic lymphohistiocytosis, IHH: Isolated hemihypertrophy, LFS: Li-Fraumeni Syndrome, MEN: Multiple endocrine neoplasia Syndrome, NBS: Nijmegen breakage Syndrome, NF1: Neurofibromatosis type I, RTPS: Rhabdoid tumor predisposition Syndrome, VHL: Hippel-Lindau Syndrome

**Cancer predisposition syndrome (CPS)**	**No. of cases**	**Neoplasms diagnosed in CPS-positive patients defined by ICD-10 classification**																							
		**C22**	**C30**	**C37**	**C38**	**C40**	**C41**	**C47**	**C48**	**C49**	**C49 | C64**	**C49 | C92**	**C56**	**C64**	**C71**	**C72**	**C73**	**C74**	**C75**	**C80**	**C81**	**C83**	**C85**	**C91**	**C92**
BWS	10	2								1				7											
BRCA1 gene mutation	1																							1	
CMMRD	2																							2	
DICER1 gene mutation	2									1				1											
Down Syndrome	19																							11	8
Edwards' Syndrome	1	1																							
FA + BRCA2 mutation	1										1														
Features of dysmorphia	3									1															2
HSCR	1							1																	
HLH	5																						1	4	
IHH	18													18											
IMAGE Syndrome	1													1											
LFS	34	5	1	1		1		7				2			13						1	1		2	
LFS + NBS	1												1												
MEN	4					1												3							
NBS	2																			1				1	
NF1	83	2			1			39	1	5					31	1	1	1	1						
Noonan Syndrome	1														1										
RTPS	2						1			1															
VHL	1														1										
WAGR Syndrome	1													1											
**No. in total**	**193**	**10**	**1**	**1**	**1**	**2**	**1**	**47**	**1**	**9**	**1**	**2**	**1**	**28**	**46**	**1**	**1**	**4**	**1**	**1**	**1**	**1**	**1**	**21**	**10**
